# Emerging Role of Multiparametric MRI in the Staging of Bladder Cancer: Insights From the BladderPath Trial

**DOI:** 10.7759/cureus.88623

**Published:** 2025-07-23

**Authors:** Daniel Akintelure, Simon Akintelure, Hasan A Al-Ibraheem

**Affiliations:** 1 Urology, Royal Gwent Hospital, Newport, GBR; 2 College of Medicine, All Saints University School of Medicine, Roseau, DMA

**Keywords:** bladder cancer, multiparametric mri, muscle invasive bladder cancer, urologic oncology, vi-rads

## Abstract

This narrative review synthesizes the current evidence on multiparametric magnetic resonance imaging (mpMRI) in bladder cancer staging, emphasizing the clinical significance of the BladderPath trial, a prospective randomized comparison of mpMRI versus conventional transurethral resection of bladder tumors (TURBT). While TURBT remains the standard diagnostic approach, emerging evidence suggests that mpMRI-based pathways may offer advantages in terms of diagnostic speed and staging accuracy. The BladderPath trial reported a median 45-day reduction in the time to definitive treatment with an imaging-first approach without compromising early oncological outcomes. However, these findings must be interpreted with caution because of the trial’s limitations, including its restrictive inclusion of patients with suspected muscle-invasive disease. This review contextualizes mpMRI within the broader diagnostic landscape, evaluates its readiness for wider clinical adoption, and outlines key challenges, such as implementation variability, access disparities, and the need for long-term data for informed evidence-based practices.

## Introduction and background

Bladder cancer represents a significant global health burden, necessitating accurate and timely staging for optimal management and treatment planning. It is one of the most prevalent and challenging urological malignancies worldwide. In 2020, there were an estimated 573,000 new cases and 213,000 deaths globally [1]. Notably, global incidence rates have risen steadily over the past two decades, particularly in aging populations and areas with high tobacco consumption, such as Eastern Europe and parts of Asia [2]. This upward trend underscores the growing importance of enhancing diagnostic efficiency and precision to improve outcomes and optimize resource utilization in the future.

The current gold-standard diagnostic pathway begins with white-light cystoscopy, followed by transurethral resection of bladder tumor (TURBT) and histological analysis to determine the depth of invasion and tumor aggressiveness. Although these procedures provide histological confirmation, they are not without limitations. White-light cystoscopy often fails to detect flat lesions, such as carcinoma in situ (CIS), and cannot provide reliable information regarding the depth of invasion [3]. TURBT understages muscle invasion in up to 30% of cases, often necessitating repeat resections [4]. TURBT is known to cause bleeding and bladder perforations, accounting for 6.5% of cases, which subsequently leads to delays in the initiation of definitive treatment, particularly in muscle-invasive bladder cancer (MIBC) [5]. These procedural risks and diagnostic inaccuracies highlight the need for non-invasive, reliable staging alternatives, such as mpMRI.

Emerging imaging modalities, particularly mpMRI using the Vesical Imaging-Reporting and Data System (VI-RADS), have shown promise in offering a non-invasive and accurate assessment of bladder wall invasion [6]. mpMRI combines multiple imaging sequences to assess anatomical details, tissue cellularity, and vascular properties. The VI-RADS scoring system standardizes the interpretation, helping to distinguish between non-muscle-invasive (NMIBC) and muscle-invasive diseases.

To address the limitations of TURBT, mpMRI offers significant benefits in the diagnosis and management of bladder cancer, particularly given the increased incidence of this disease. One of the key advantages of mpMRI is its ability to provide detailed anatomical and functional imaging, which aids in the accurate tumor staging and evaluation of muscle invasiveness in bladder cancer. VI-RADS was developed to standardize mpMRI acquisition and interpretation, which improves inter-reader agreement and enhances the reliability of mpMRI in clinical practice [7]. mpMRI is particularly useful for differentiating MIBC from NMIBC. This differentiation is crucial because it affects treatment approaches. The ability of mpMRI to accurately stage bladder cancer is notable, as traditional staging methods often lead to understaging because of the underestimation of tumor depth and nodal involvement. mpMRI has shown high sensitivity and specificity in detecting MIBC and regional nodal disease, offering a non-invasive and accurate alternative to current diagnostic practices [8,9]. mpMRI contributes to a scalable and efficient diagnostic pathway by providing high diagnostic accuracy and allowing better risk stratification, which optimizes treatment selection [10].

Despite these benefits, several challenges are associated with the implementation of mpMRI in clinical practice. One of the key challenges is ensuring the consistent execution and high-quality reporting of mpMRI scans. The quality and reliability of mpMRI results can vary based on radiologist expertise and the protocols used [11]. In addition, the interpretation of mpMRI data is complex and can be time-consuming, posing challenges in maintaining sensitivity and specificity. Manual interpretation requires significant expertise, which may not be universally available [12]. Another significant challenge is the cost-effectiveness of mpMRI; the high cost of the technology and the training required for accurate interpretation can limit its widespread adoption, particularly in resource-limited settings [11,13].

The BladderPath trial was a prospective, multicenter, randomized controlled study conducted in the United Kingdom to evaluate whether an imaging-first diagnostic pathway using mpMRI could safely and efficiently triage patients with suspected MIBC [14]. A total of 219 patients were enrolled based on specific inclusion criteria: newly diagnosed bladder tumors visible on flexible cystoscopy and clinical suspicion of MIBC. Participants were randomized to either a standard care pathway (TURBT followed by imaging and definitive treatment) or an imaging-first pathway using VI-RADS-based mpMRI and targeted biopsy. The primary endpoint was time from diagnosis to definitive treatment. The study demonstrated a median 45-day reduction in time to treatment initiation in the imaging-first group (53 days vs. 98 days), with no compromise in short-term oncologic outcomes. mpMRI was interpretable in over 90% of cases. However, limitations included the restricted study population, which excluded NMIBC, a relatively short follow-up period (14 months), and potential variability in radiologic interpretation and biopsy technique across centres. The BladderPath trial presents the most compelling prospective evidence supporting a shift in diagnostic pathways from resection-first to imaging-first for newly diagnosed bladder tumors [14]. This aligns with the broader movement toward precision medicine, in which individualized risk assessment informs tailored interventions. While several reviews have explored the use of mpMRI in bladder cancer, few have critically evaluated its readiness to assume a central role in clinical practice, particularly in light of recent high-quality trial data.

This review synthesizes the current evidence, including the BladderPath trial, to argue that mpMRI represents a viable and potentially superior approach for bladder cancer staging. By enhancing diagnostic precision and reducing procedural risks and treatment delays, mpMRI-based pathways may soon redefine the standards of care.

## Review

Historical context and limitations of TURBT

TURBT has been the cornerstone of bladder cancer staging since the mid-20th century. Initially introduced as both a diagnostic and therapeutic tool, TURBT was heralded for its ability to directly visualize and sample the bladder lesions. Over the decades, refinements such as en bloc resection (ERBT), photodynamic diagnosis (PDD), and bipolar resection systems have been attempted to enhance the resection quality and reduce complications.

Despite these advances, there are significant limitations. Approximately 25-30% of MIBC cases are initially understaged, which can delay curative treatment and increase the risk of disease progression and mortality [15]. Repeat resections are frequently needed for accurate staging, reported in up to 30% of cases, particularly when the initial resection is incomplete or the pathology is ambiguous [16]. Procedural complications include bleeding (5-10%), bladder perforation (up to 5%), and anesthesia-related risks, particularly in older patients with comorbidities [17]. 

In addition, its comparative efficacy versus mpMRI remains limited. For example, the PHOTO randomized trial by Heer et al. demonstrated that although PDD significantly improves tumor detection, it did not significantly reduce long-term recurrence rates compared to white light cystoscopy, with a recurrence hazard ratio of 0.94 (95% CI, 0.69 to 1.28; P = 0.70) and a three-year recurrence-free survival of 57.8% for PDD versus 61.6% for white light cystoscopy [18]. Moreover, there was no evidence of cost-effectiveness or quality-of-life benefit, with a quality-adjusted life year difference of -0.007 (95% CI, -0.133 to 0.119; P = 0.444) [18]. Likewise, a meta-analysis by Yanagisawa et al. reported that ERBT reduced the risk of bladder perforation (RR 0.13; 95% CI 0.05-0.34) and improved detrusor muscle presence in specimens (RR 1.31; 95% CI 1.19-1.43), but did not significantly lower recurrence rates at 12 and 24 months [19]. By contrast, mpMRI with VIRADS showed superior diagnostic accuracy. A prospective validation study reported a sensitivity of 91.9%, specificity of 91.1%, and AUC of 0.94 in discriminating NMIBC from MIBC, with strong inter-reader agreement (k = 0.81) [20]. These data indicate that while TURBT enhancements improve surgical quality, mpMRI offers more non-invasive staging for reliable and accurate pre-treatment decision-making.

Moreover, delays associated with scheduling surgery, awaiting pathology, and performing follow-up procedures can lead to median treatment initiation times of >90 days in many healthcare settings. Such delays are associated with worse survival outcomes, particularly in patients with rapidly progressing MIBC [17]. These systemic challenges have spurred interest in alternative approaches that provide earlier, safer, and more accurate staging information.

Role of mpMRI and development of VI-RADS

 mpMRI integrates three core sequences: T2-weighted imaging (T2WI), diffusion-weighted imaging (DWI), and dynamic contrast-enhanced imaging (DCE) [6]. T2WI provides high-resolution anatomical details, allowing the visualization of the bladder wall layers. In NMIBC, a low-signal-intensity tumor typically abuts but does not disrupt the high-signal-intensity muscularis propria. In contrast, MIBC is characterized by disruption or invasion of the muscularis layer. DWI reflects tissue cellularity. Tumors typically show restricted diffusion due to their high cellular density. In MIBC, more extensive and deeper diffusion restrictions are observed, indicating transmural invasion. DCE imaging evaluates tumor vascularity by tracking contrast enhancement patterns. MIBC lesions often demonstrate early and intense enhancement, whereas NMIBC shows superficial and localized contrast uptake.

The VI-RADS scoring system standardizes the mpMRI interpretation. Scores of 1 or 2 indicate a low likelihood of muscle invasion, whereas scores of 4 or 5 suggest a high likelihood. A score of 3 is considered equivocal and often necessitates further evaluation [6].

VI-RADS streamlines mpMRI interpretation with standardized scoring and has shown high diagnostic accuracy (sensitivity 88.2%; specificity 80.6%) with substantial inter-reader agreement (mean k = 0.82) across studies [21]. However, interpretation may be less reliable in low-experience settings because of several challenges, such as limited access to high-quality hardware, inconsistent training in bladder MRI protocols, and the absence of routine double-reading workflows. Compared to TURBT, which can underestimate muscle invasion in up to 30% of cases, mpMRI offers more consistent preoperative risk stratification. However, inter-reader variability remains a concern, particularly in lower-experience settings such as ours. Initiatives to mitigate this include training modules, consensus guidelines, and double-reading protocols.

The BladderPath trial: a paradigm shift

The BladderPath trial, published in the Journal of Clinical Oncology in 2024, was a prospective, multicenter, randomized controlled trial conducted in the UK [14]. A total of 219 patients with newly diagnosed and imaging-suspected MIBC were enrolled. The inclusion criteria were a visible bladder tumor on flexible cystoscopy and a high likelihood of MIBC based on the initial assessment. Participants were randomized to either the standard pathway (TURBT followed by imaging) or an imaging-first pathway using multiparametric MRI (mpMRI) and targeted biopsy.

The key methodological features are as follows: The study involved a sample size of 219 patients recruited from multiple centers. The primary outcome was the time from diagnosis to the initiation of definitive treatment. Secondary outcomes included diagnostic accuracy, safety, and clinical outcomes at follow-up. Statistical analysis employed an intention-to-treat design, with hazard ratios and Kaplan-Meier curves used to evaluate time-to-treatment outcomes.

Findings showed that the median time to definitive treatment (such as cystectomy or chemoradiation) was 45 days shorter in the mpMRI-first arm than in the standard pathway (53 days vs. 98 days). No significant differences were observed in recurrence rates or early oncologic outcomes between the groups. The median follow-up time was 14 months, which, although relatively short, was sufficient to assess early oncologic outcomes such as recurrence and progression. mpMRI was deemed feasible and interpretable in >90% of cases. In addition, the study reported a reduction in resource use, particularly in terms of repeat TURBTs and operating room time.

However, this study had some limitations. It included only patients suspected of having MIBC, which limits its applicability to broader populations, particularly those with NMIBC. Potential biases include operator-dependent interpretation of imaging and variability in the biopsy techniques across centers. Moreover, long-term oncological outcomes were not available at the time of this publication. Despite these limitations, the trial supports the feasibility and clinical safety of imaging-first approaches and highlights their potential to streamline patient care.

While the BladderPath trial provides robust prospective evidence for an imaging-first approach to bladder cancer staging, its external validity warrants careful scrutiny. The trial was conducted within the UK National Health Service (NHS), which benefited from a centralized infrastructure, universal health coverage, and coordinated care pathways that facilitated timely mpMRI access, standardized radiological interpretation, and streamlined follow-up [14]. These conditions may not be easily replicated across other healthcare systems, particularly in settings with constrained imaging infrastructure, fragmented referral networks, or variable clinical governance. In many low- and middle-income countries (LMICs), key implementation barriers include limited access to high-resolution MRI, a shortage of radiologists trained in urogenital mpMRI, and the absence of formal VI-RADS training or quality assurance programs [22]. Even in high-income countries, disparities between tertiary academic centers and community hospitals can lead to heterogeneity in imaging quality and diagnostic interpretations [23]. Reimbursement models also influence feasibility; while publicly funded systems, such as the NHS, may support imaging-led pathways as a cost-effective strategy, fee-for-service frameworks may inadvertently disincentivize non-procedural diagnostics, such as mpMRI. Moreover, the patient population enrolled in the BladderPath trial may not fully reflect the global epidemiological patterns. For example, regions with endemic schistosomiasis or a higher prevalence of squamous cell carcinoma exhibit different tumor biology and imaging characteristics, potentially limiting the generalizability of the VI-RADS-based staging [24]. Consequently, while the BladderPath trial offers a critical foundation for practice change, widespread adoption will require health system-level adaptations, scalable training solutions, and validation across diverse clinical settings. 

Comparative literature and global perspective

Several studies have corroborated the diagnostic utility of mpMRI for staging bladder cancer. For example, Takeuchi et al. demonstrated the high sensitivity of DWI for T staging and grading, with sensitivities exceeding 85% for MIBC detection [25]. Del Giudice et al. validated the clinical utility of the VI-RADS, which improved the triage and management of high-risk NMIBC, potentially avoiding unnecessary interventions [20]. However, it is crucial to note that many of these studies applied VI-RADS retrospectively, which may limit their generalizability to real-world clinical workflows. Retrospective use often benefits from ideal image quality and post hoc expert consensus, which may not reflect their variability and resource constraints in prospective, routine implementation. By contrast, the BladderPath trial applied VI-RADS prospectively as part of an imaging-first diagnostic strategy, offering stronger evidence of its feasibility and reliability in real-world settings [14]. Future prospective studies across diverse healthcare systems are needed to validate the VI-RADS in lower-resource or lower-experience contexts and to inform widespread adoption.

Systematic reviews by Woo et al. and Feng et al. affirmed the diagnostic superiority of mpMRI over conventional modalities, particularly in distinguishing NMIBC from MIBC [26,27]. These reviews included studies across various populations and imaging protocols, enhancing the generalizability of their results.

However, its global adoption remains uneven. High-income countries in Europe and North America are beginning to integrate mpMRI into bladder cancer algorithms, guided in part by EAU recommendations [3]. By contrast, LMICs face considerable barriers, including limited MRI infrastructure, a shortage of trained radiologists, and cost constraints. For example, recent data highlight stark disparities in MRI availability: high-income countries report a mean of approximately 27 MRI units per million population, whereas upper-middle-income countries average only 5.4 units per million population and lower-middle-income countries average approximately 1.1 units per million population (even as low as 0.2-0.4 units per million population with low-field strength systems in low-income contexts) [28]. Concurrently, radiologist density mirrors this inequity: sub-Saharan Africa has approximately 0.9 radiologists per million population, compared to 47-110 radiologists per million population in Europe [29]. These capacity gaps suggest that even if mpMRI protocols such as VI-RADS are adopted, their effectiveness may be significantly constrained in LMIC settings due to a lack of infrastructure, limited equipment maintenance support, and a shortage of trained specialists. Moreover, reimbursement frameworks in many health systems still prioritize procedural interventions (e.g., TURBT) over diagnostic imaging. Unless systems invest in scalable equipment, training modules, and quality assurance, the benefits of BladderPath may not be universally achievable.

Future directions and implementation considerations

To integrate mpMRI into routine clinical pathways, structured models should be developed, drawing inspiration from analogous shifts in prostate cancer, where MRI-first approaches are the standard.

The key components of successful implementation include several critical elements. First, radiologist training in VI-RADS and standardized interpretation protocols is essential to ensure consistent and accurate assessment of mpMRI findings. Second, clinical decision pathways must be developed that effectively integrate mpMRI results with biopsy data and relevant biomarker information to guide patient management strategies. Third, cost-effectiveness analyses are vital, including ongoing trials such as the economic sub-studies of the BladderPath trial. These analyses should account for both direct healthcare costs, such as reductions in the number of procedures and operating room time, and indirect costs, including shorter time to treatment and fewer complications. Finally, integration with molecular diagnostics, including urinary DNA- or RNA-based tests, may enhance the risk stratification. For example, Lokeshwar et al. demonstrated the potential utility of non-cytology-based biomarkers that could complement imaging approaches [30].

In the short term, implementation efforts may be most effective when focused on high-volume or high-risk tertiary referral centers where radiologic and urologic expertise is already established. These centers can serve as regional hubs for training, pilot programs, and data collection. Over time, broader integration into general urological practice may be supported by scalable training modules, remote reading solutions, and decision-support tools that facilitate standardized reporting. Long-term strategies should include national guideline endorsement, equitable MRI infrastructure investment, and integration into electronic health systems to enable seamless triage, staging, and longitudinal care pathways across a wider range of clinical environments. 

Frameworks should also account for regional variations in MRI availability. Hybrid strategies, such as the selective use of mpMRI in high-risk or equivocal cases, could be a pragmatic interim solution.

## Conclusions

The BladderPath trial provides robust evidence that mpMRI, when used in a standardized imaging-first pathway, can accelerate the diagnostic timeline and safely triage patients with suspected MIBC. While these findings support the feasibility of mpMRI as a triaging tool, its broader integration into standard care will depend on its reproducibility across diverse settings and patient populations.

Rather than suggesting a full replacement of TURBT, current evidence supports mpMRI as a complementary modality, particularly valuable in high-risk patients or those with clinical features suggestive of MIBC. Implementation should begin in high-resource centers with the capacity for radiologic standardization and multidisciplinary coordination. Key criteria for mpMRI-first triage may include visible solid tumors on flexible cystoscopy, hematuria with a high clinical suspicion, or equivocal findings on initial imaging.

Future guideline updates should reflect this nuanced role, recommending mpMRI where infrastructure and expertise exist, while also supporting hybrid diagnostic strategies. Further validation studies, especially those examining long-term oncologic outcomes, cost-effectiveness, and applicability in low-resource settings, are crucial to defining the sustained role of mpMRI in bladder cancer staging. Thus, while BladderPath findings are promising, widespread adoption should proceed with clinical caution and structured evaluation frameworks.
